# Supplementary material for: GraphDDP: a graph-embedding approach to detect differentiation pathways in single-cell-data using prior class knowledge
Source: Nat Commun. 2018 Sep 11;9:3685. doi: 10.1038/s41467-018-05988-7 (PMC6134144; doi:10.1038/s41467-018-05988-7)
Supplement: Supplementary file 1 — Supplementary Information [file 41467_2018_5988_MOESM1_ESM.pdf]

Supplementary Note 1

Experimental parametric setup.

GraphDDP uses several parameters to control pre-processing, the neighborhood relation and the confidence in the given clustering. For the pre-processing (see Fig. 1a and b), the first parameter is `class filtering`, which is the threshold for not displaying clusters in the layout that contain only very few cells. It is used to remove noisy cluster annotations. When `normalization` is set to `True`, all cells are represented as vectors that have unitary norm, i.e., the read counts are transformed into relative proportions. If `feature selection` is `True`, then a feature (i.e., the counts associated to specific gene) is discarded if it does not improve the multi-class accuracy of a SVM classifier (as described in Methods). When `feature transformation` is `True`, then each true cell i is represented as feature vectors of size $n =$ total number of cells in the dataset. Each entry j in the vector contains the correlation coefficient of the profile expression of cell i w.r.t. cell j.

These parameters determine the pre-processing of the data. The construction of the graph, which is then visualized in the layout (see Fig. 1)c and d), is parametrized by: `num nearest neighbors (k)` the number of nearest neighbors links to same class instances, `num nearest neighbors (k')` the number of nearest neighbors links to different class instances that are denser and `knn horizon` the maximum number of nearest neighbors considered when searching for shift neighbors.

Finally, in the layout phase (see Fig. 1e and f), the `user confidence C` is the contraction factor for the desired distance $d'(x,z)$ between instances x and z according to: $d'(x,z) = \frac{d(x,z)}{1+C}$. When $C = 0$ no contraction is done. As C increases, the clusters become more compact.

Myeloid Progenitor Data Set

We directly received the processed myeloid progenitor directly from the authors, consisting of a cluster assignment of 2,730 filtered cells to 19 clusters and a matrix $(U_{i,j})$ of UMI (unique molecular identifier) counts, where $U_{i,j}$ denotes the UMI counts for gene i and cells j. We then normalized the gene counts to the total counts per cell. As all clusters are of reasonable size we do not filter them and retain all 19 clusters. Gene counts were normalized, and feature selection was turned off as the data was already filtered, resulting in 8,716 features. We used feature transformation into correlation-based features. For the modeling, we used $k = 5$ for number of nearest neighbor edges, and $k' = 2$ for the number of shift edges. The k-NN horizon was set to 20, and the user confidence for the clustering was set to 25 (see Supplementary Table 1 for a summary of these settings).

	Parameter										
	class filtering min threshold	num classes	normal-ization	feature selection	num features	num cells	feature transf.	k nearest edges	k' shift edges	h knn horizon	C user confidence
myeloid progenitor data[21]	False	19	True	False	8,716	2,730	True	5	2	20	25
intestinal epithelial cells data[17]	5	27 → 19	True	True	1,421→281	462	True	5	1	10	1.5

Supplementary Table 1. Summary of parameter settings for the two data sets.

Code

The source code is available at: `https://github.com/fabriziocosta/GraphEmbed`. In addition we provide a Jupiter notebook[35] where the user can set all the parameters using sliding bars and observe interactively the effect on the layout.

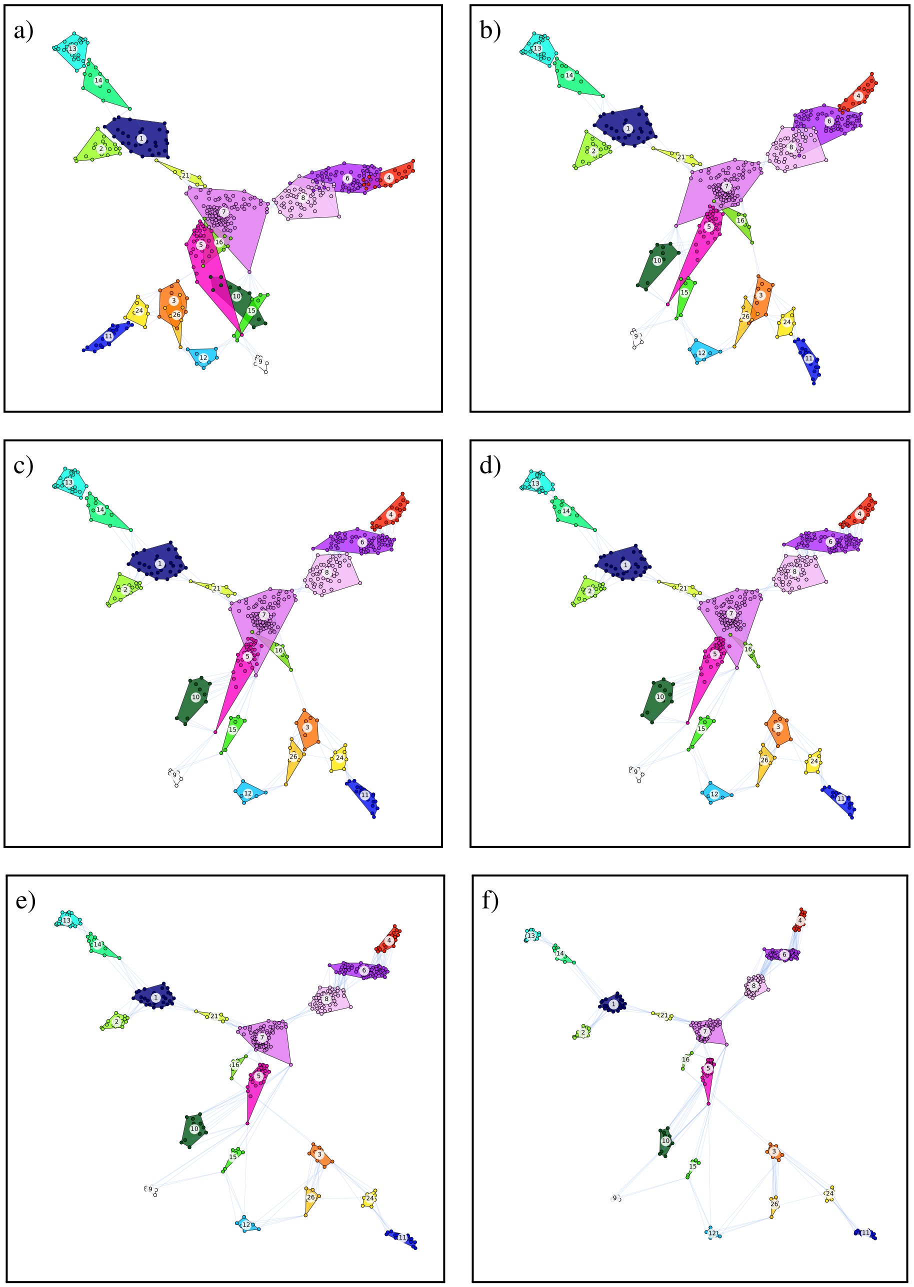

Supplementary Figure 1. GraphDDP visualization of the intestinal epithelial cells dataset[17]. The plots show the effect of increasing the user confidence parameter C. a) $C = 0$. b) $C = 0.5$. c) $C = 1$. d) $C = 1.5$. e) $C = 4$. f) $C = 8$. Clusters tends to become more compact and separated as C increases. Note that the layout reaches a stable configuration after a critical value of $C \approx 0.5$

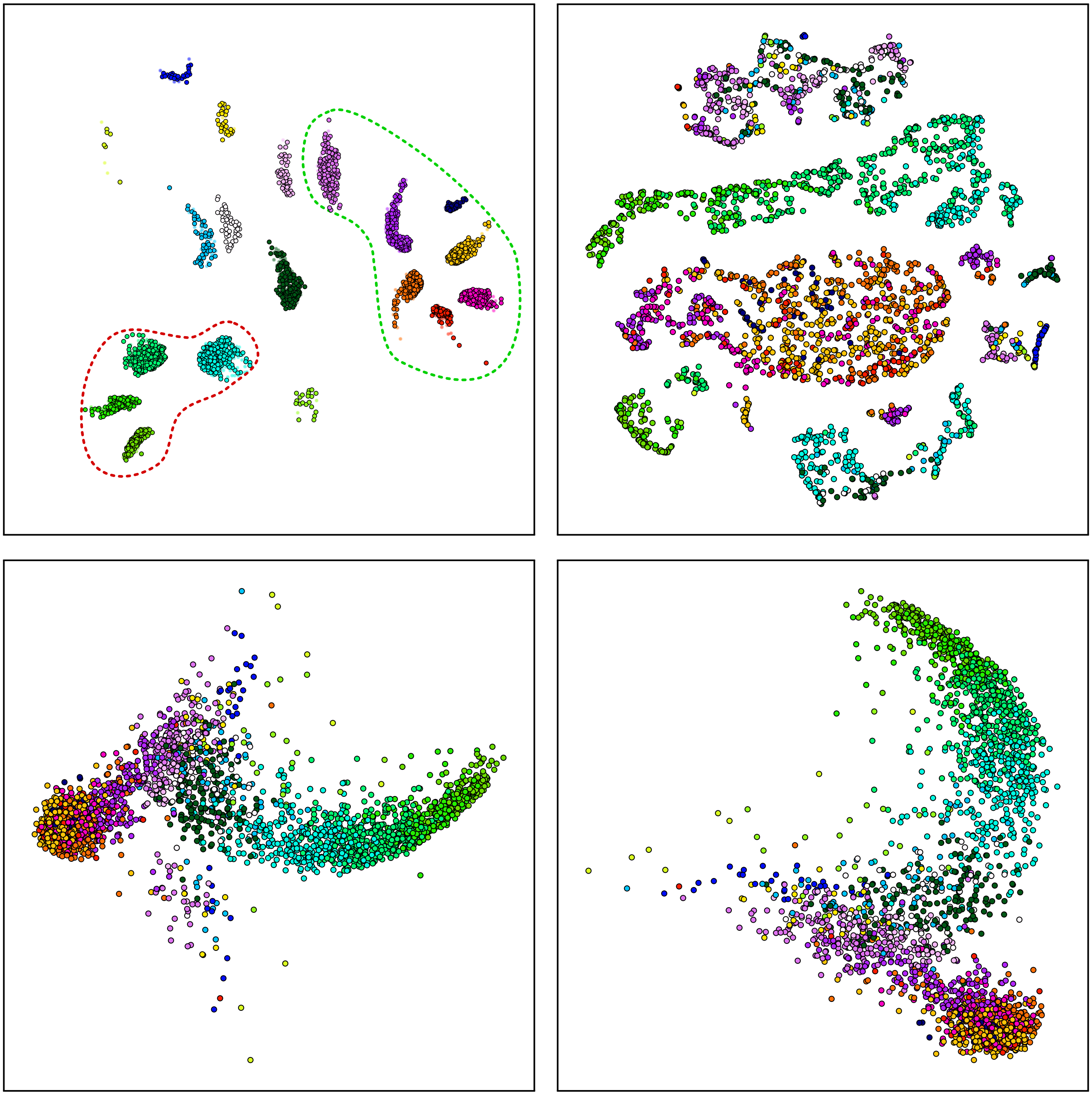

Supplementary Figure 2. Layout of the myeloid progenitors data[21] according to different visualization approaches. a) Layout as in Fig. 3 without links and convex regions. We have indicated cells that are related to neutrophil (red) and erythrocyte (green) differentiation. Due to the use of prior knowledge of classes, our GraphDDP algorithm is able to visualize both class membership (which corresponds to cell types) and differentiation trajectories (see Fig. 3 and main text for extended discussion) b) In comparison, t-SNE fails on the erythrocyte-related cell types, is only partially able to cluster neutrophil-related cells and completely fails to visualize differentiation pathways. c) and d) Both MDS and PCA completely fail to visualize clusters of cells related to common cell types, and are only partially able to visualize the neutrophil differentiation. Erythrocyte differentiation however cannot be detected.

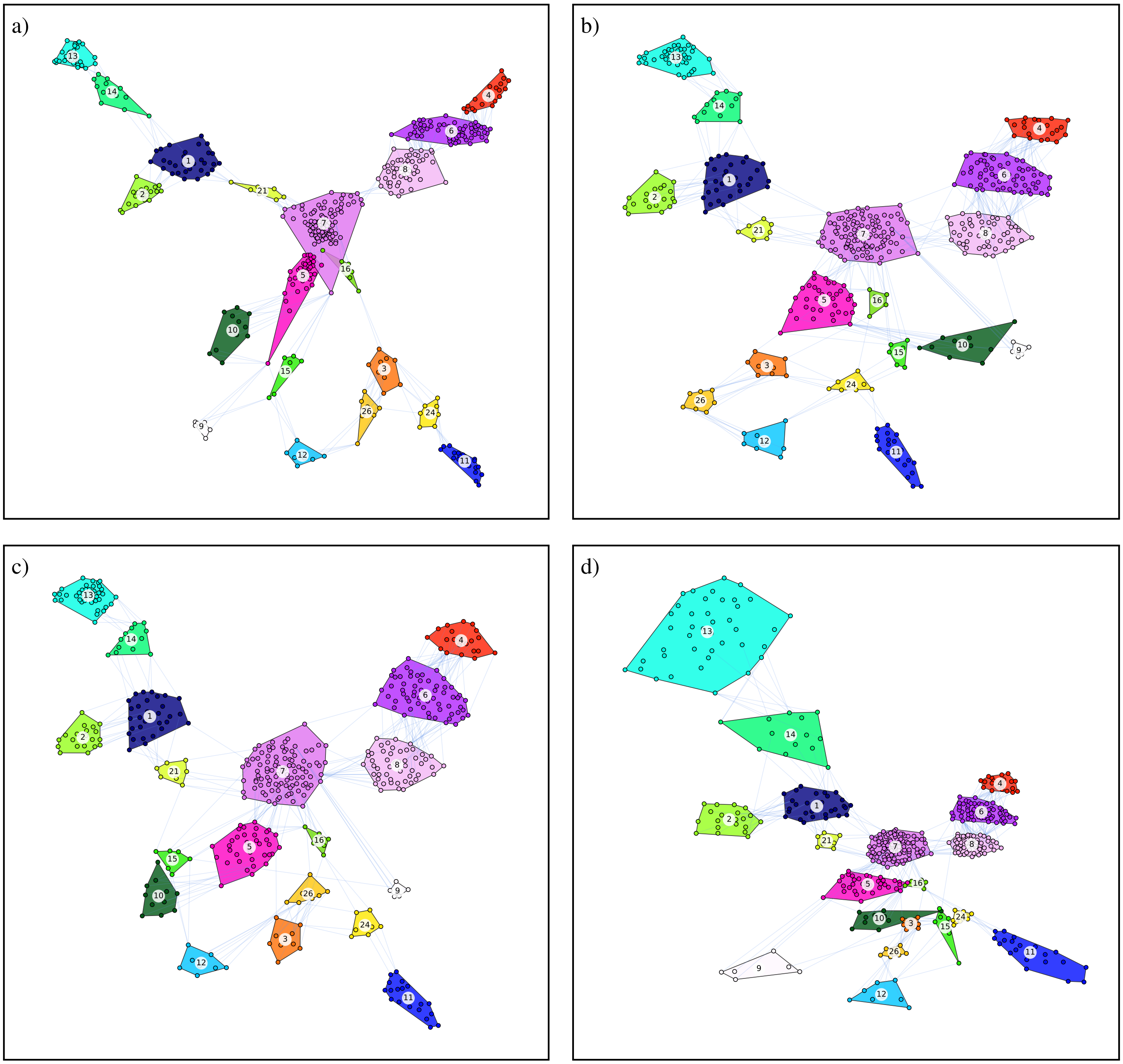

Supplementary Figure 3. GraphDDP layout with a decreasing number of pre-processing steps: a) normalization, feature selection, correlation transformation, b) normalization, feature selection, no correlation transformation, c) normalization, no feature selection, no correlation transformation, d) no normalization, no feature selection, no correlation transformation. We can notice that the grouping of clusters into differentiation trajectories is less well defined when pre-processing steps are omitted.

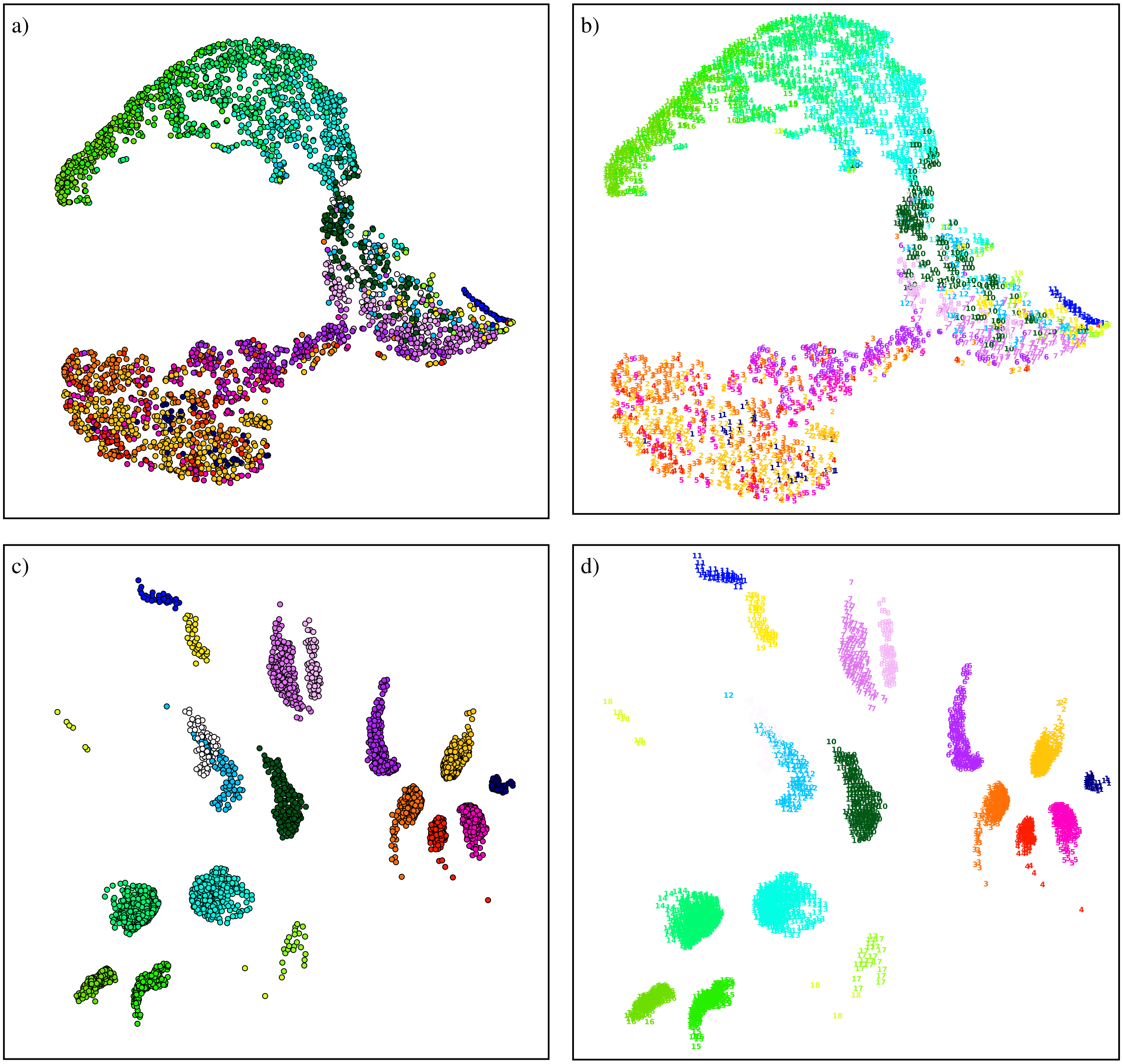

Supplementary Figure 4. Comparison between t-SNE layout and GraphDDP layout (same preprocessing) on myeloid data. Here t-SNE is run with a high perplexity in order to avoid the formation of disconnected components: as a consequence finer class separations are lost. a) t-SNE layout b) t-SNE layout with class details, c) GraphDDP layout, d) GraphDDP layout with class details. A mix of different classes can be observed especially in the lower and middle part of the layout

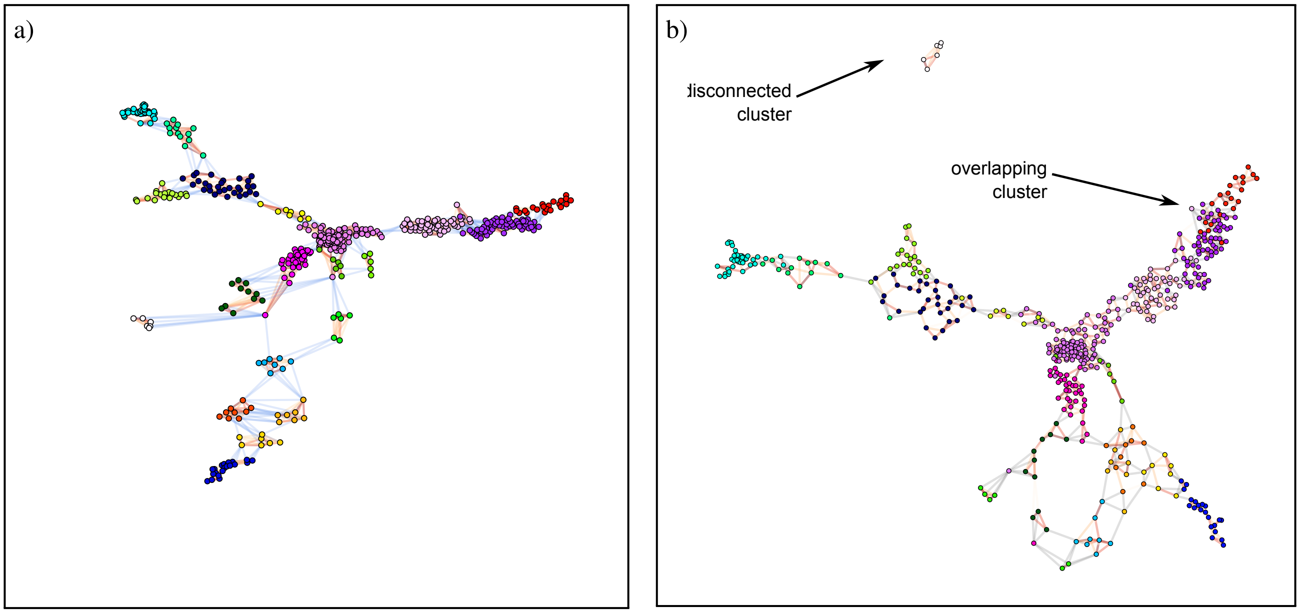

Supplementary Figure 5. Effect of neglecting k-shift edges with a weaker setting for the neighborhood constraints. a) GraphDDP layout for the intestine dataset, b) K-NN layout without k-shift edges for the intestine dataset and small k=3. The resulting layout is disconnected as there are few or no connections (see example cluster) to other clusters. In addition, as there is no contraction, one can also see overlapping clusters (one example is indicated).

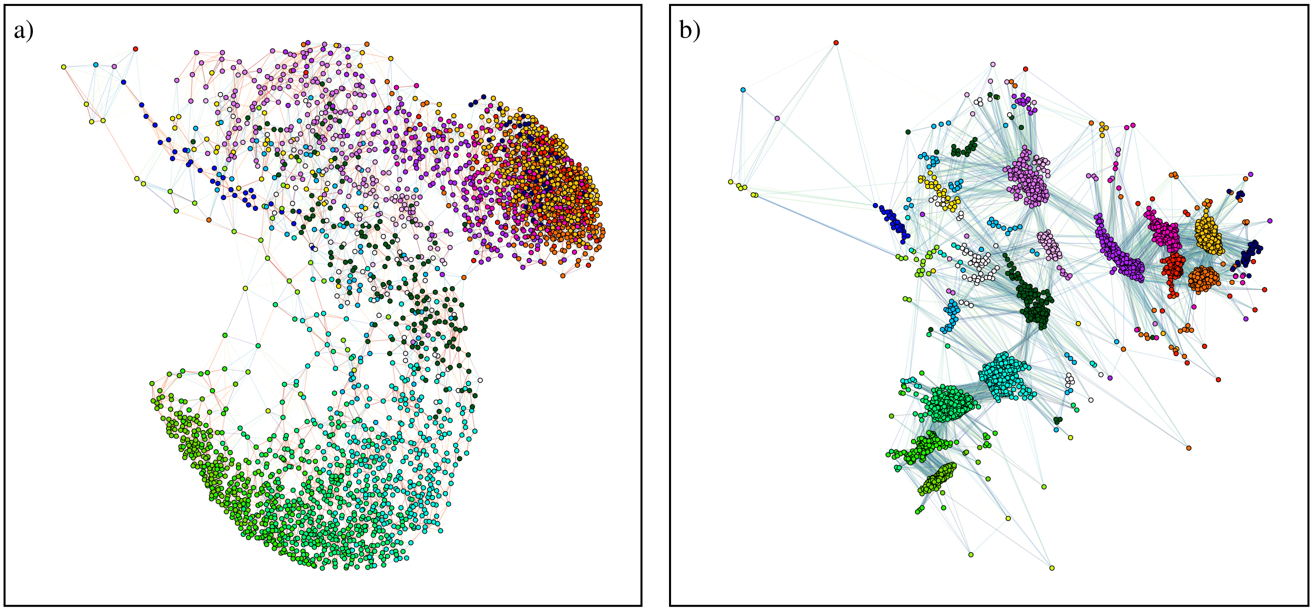

Supplementary Figure 6. Effect of neglecting k-shift edges with a stronger setting for the neighborhood constraints. Shown are two K-NN layouts without k-shift edges for the myeloid dataset and larger k=7. a) Original edge length, no contraction of cells belonging to the same class. The resulting layout is noisy and results in fragmented classes, especially in the less populated clusters; b) edge length between same class instances is strongly contracted; using the clustering as prior information does improve the resulting layout to some extent; however, it is still quite noisy, again especially for the less populated clusters.

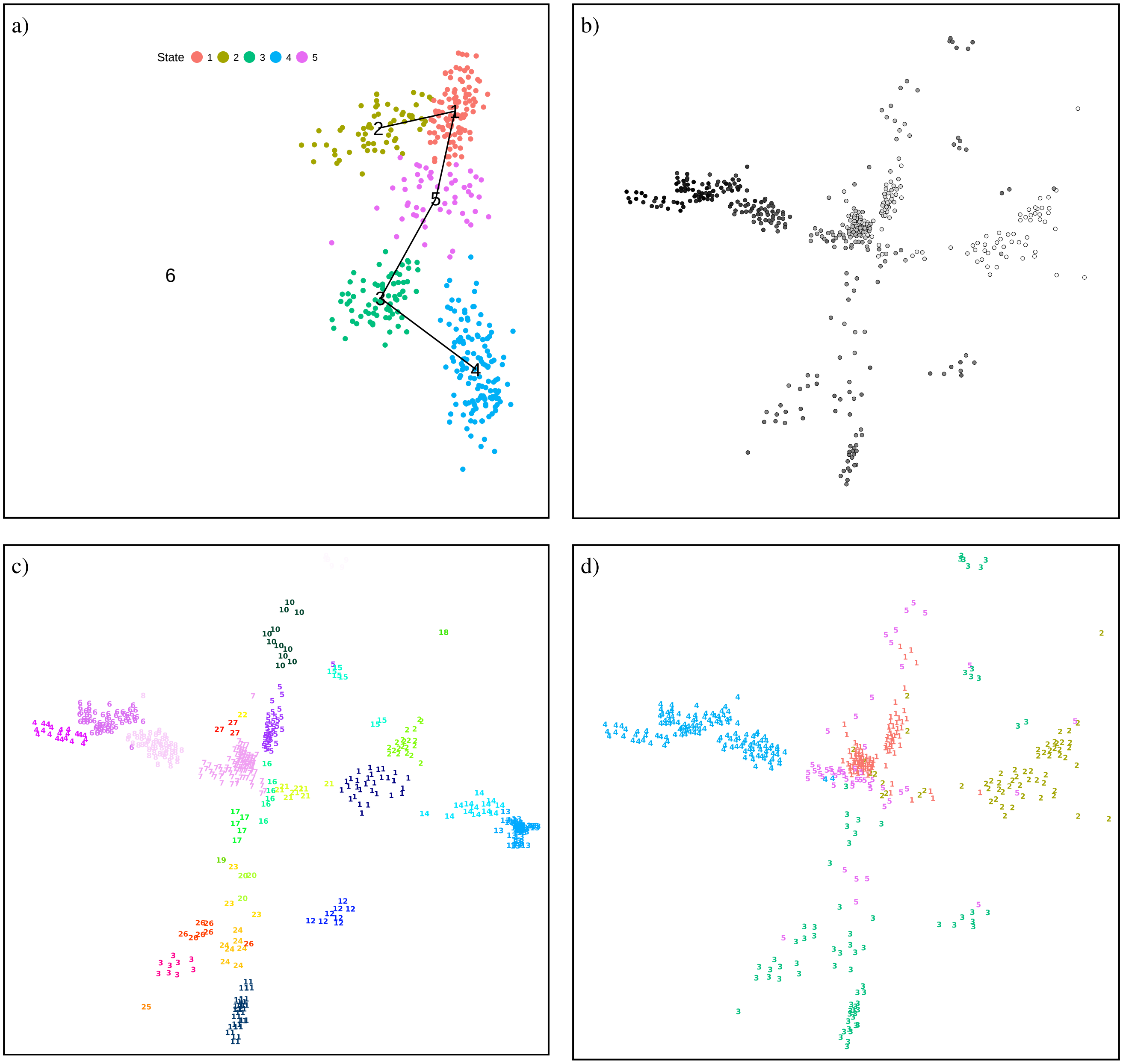

Supplementary Figure 7. Comparison with TSCAN[23] on intestine data. a) TSCAN[23] layout of the intestine data. For this data set, TSCAN accepts only a maximum of 6 classes and finds only one linear pathway $2 \rightarrow 1 \rightarrow 5 \rightarrow 3 \rightarrow 4$ on these classes. b) GraphDDP layout with gray level proportional to pseudo-time score given by TSCAN; the pseudotime has more or less a vertical direction (from black on the left to white on the right), and all the cells in middle axis do not have a clear pseudotime ordering. c) GraphDDP layout with class details given by the user defined clustering, d) GraphDDP layout with class details as identified by TSCAN; there are several mixed classes, especially for the smaller clusters.

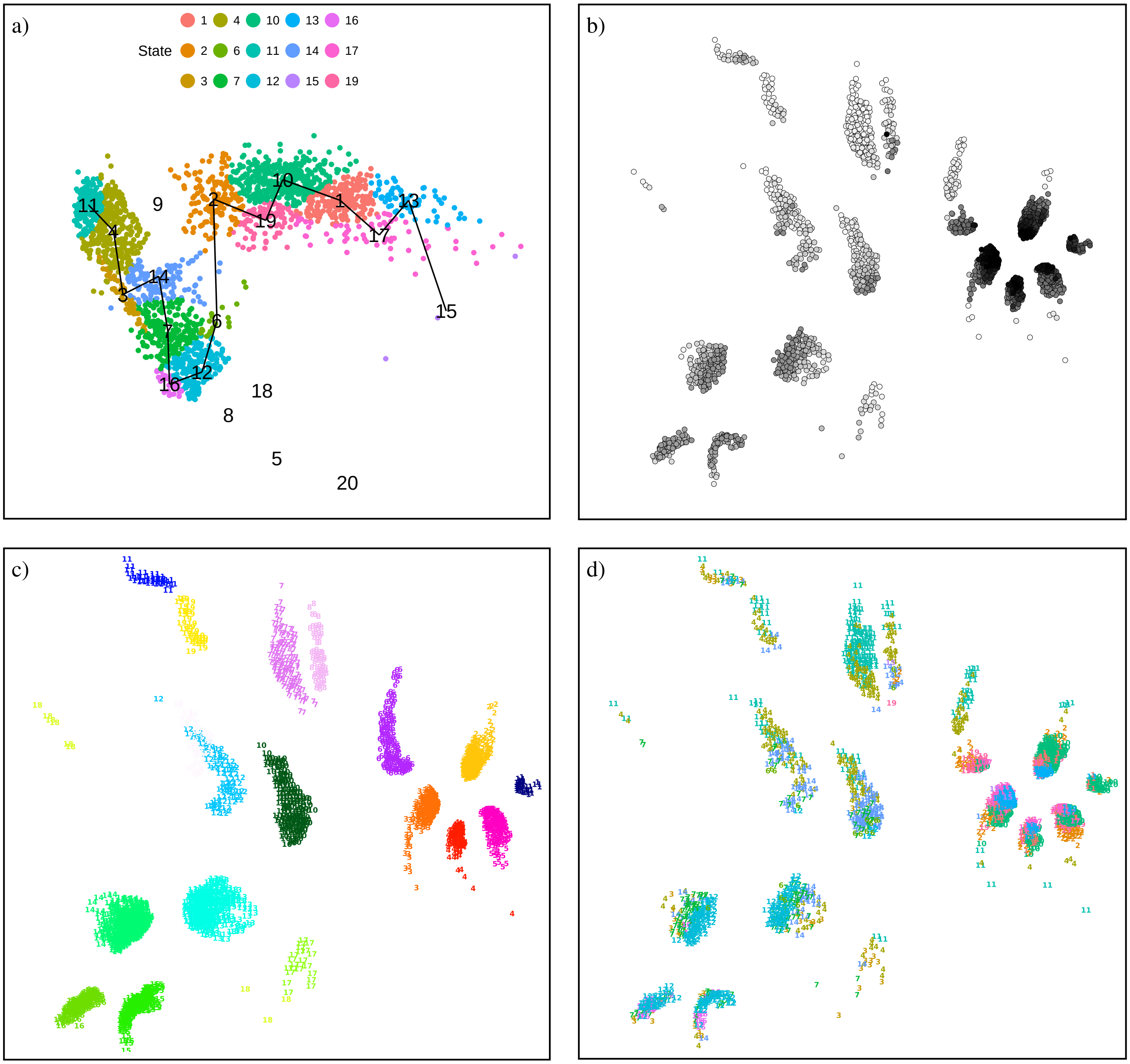

Supplementary Figure 8. Comparison with TSCAN on myeloid data. a) TSCAN layout; TSCAN detects one differentiation pathway; b) when displaying the TSCAN determined pseudotime on our GraphDDP layout (gray level proportional to TSCAN pseudo-time score), it can be seen that the pseudotime does not clearly show major differentiation pathways; instead, it seems that the pseudotime is more pronounced within the clusters provided by the original publication[21]. c) GraphDDP layout with class details (i.e., the given by clustering from[21]), d) GraphDDP layout with class details identified by TSCAN; it also shows that the classes are spread over the user-defined clustering.

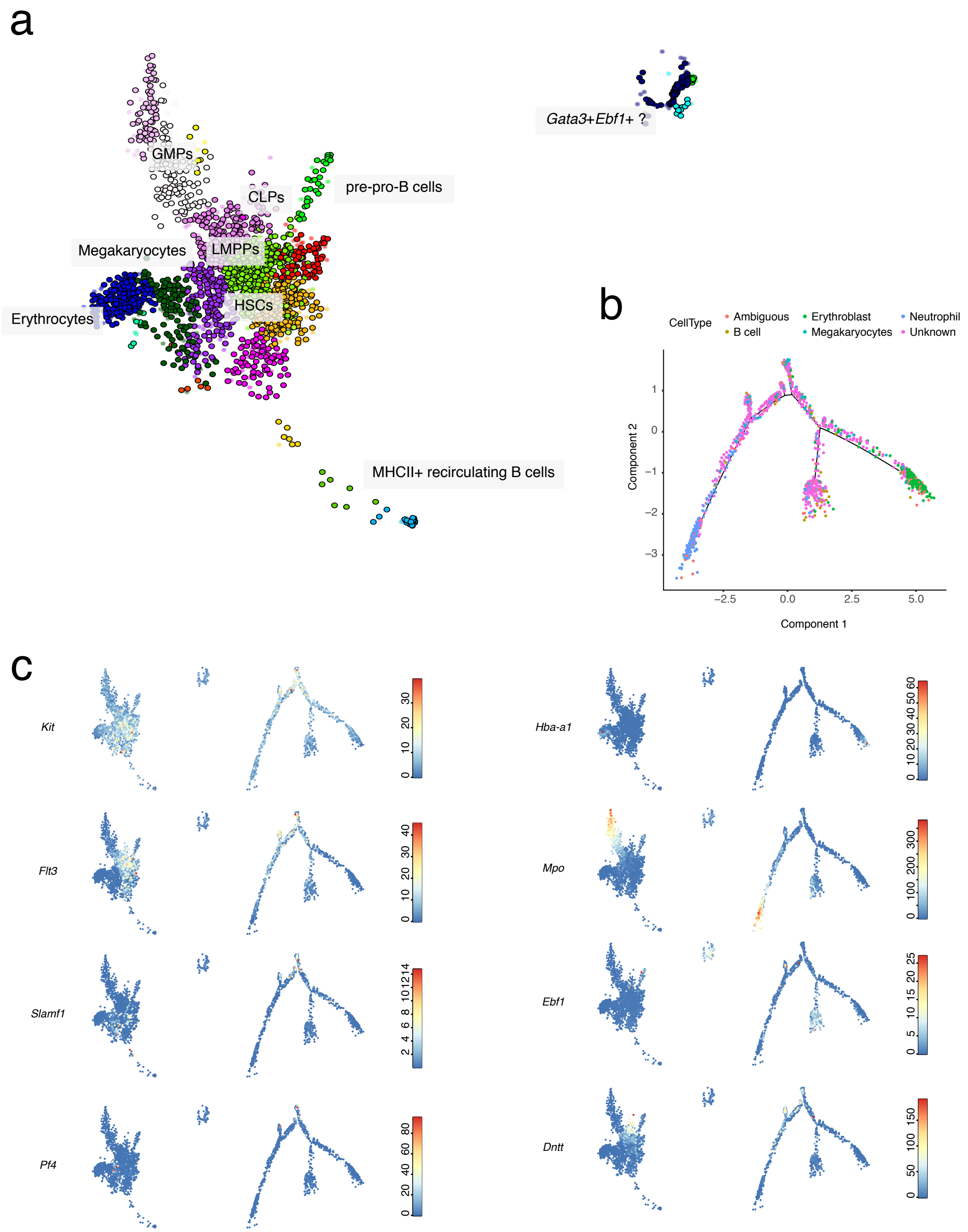

Supplementary Figure 9. Comparison of GraphDDP and Monocle2[28] on murine hematopoietic progenitors. a) GraphDDP was run on single-cell RNA-seq data of murine hematopoietic progenitors[26] with RaceID3[27] clusters as input. The layout reflects the known differentiation hierarchy with progenitor populations identified based on known markers. Hematopoietic stem cells (HSCs): *Kit+Ly6a+Slamf1+*; lymphoid primed multipotent progenitor (LMPP): *Kit+Ly6a+Flt3+*; common lymphoid progenitor (CLP): *Kit*^{low}*Ly6a*^{low}*Il7r+*; megakaryocytes: *Pf4+*; erythrocytes: *Hba-a1+*; granulocyte macrophage progenitors: *Mpo+*; B cells: *Ebf1+*. b) Monocle2 analysis of the same dataset as in (a). c) Gene expression maps for marker genes of the different sub-populations. *Dntt* is shown as a highly expressed marker of CLPs. For each gene, the left panel shows gene expression in the GraphDPP layout, and the right panel depicts the corresponding map obtained by Monocle2. The color legend indicates normalized gene expression obtained from the original study.

Supplementary References

1. Islam, S. *et al.* Characterization of the single-cell transcriptional landscape by highly multiplex RNA-seq. *Genome Res* **21**, 1160–7 (2011). DOI 10.1101/gr.110882.110.
2. Jaitin, D. A. *et al.* Massively parallel single-cell RNA-seq for marker-free decomposition of tissues into cell types. *Science* **343**, 776–9 (2014). DOI 10.1126/science.1247651.
3. Treutlein, B. *et al.* Reconstructing lineage hierarchies of the distal lung epithelium using single-cell RNA-seq. *Nature* **509**, 371–5 (2014). DOI 10.1038/nature13173.
4. Grün, D. *et al.* Single-cell messenger RNA sequencing reveals rare intestinal cell types. *Nature* **525**, 251–5 (2015). DOI 10.1038/nature14966.
5. Stegle, O., Teichmann, S. A. & Marioni, J. C. Computational and analytical challenges in single-cell transcriptomics. *Nat Rev Genet* **16**, 133–45 (2015). DOI 10.1038/nrg3833.
6. Grün, D. & van Oudenaarden, A. Design and Analysis of Single-Cell Sequencing Experiments. *Cell* **163**, 799–810 (2015). DOI 10.1016/j.cell.2015.10.039.
7. Zeisel, A. *et al.* Brain structure. Cell types in the mouse cortex and hippocampus revealed by single-cell RNA-seq. *Science* **347**, 1138–42 (2015). DOI 10.1126/science.aaa1934.
8. Xu, C. & Su, Z. Identification of cell types from single-cell transcriptomes using a novel clustering method. *Bioinformatics* **31**, 1974–80 (2015). DOI 10.1093/bioinformatics/btv088.
9. Guo, M., Wang, H., Potter, S. S., Whitsett, J. A. & Xu, Y. SINCERA: A Pipeline for Single-Cell RNA-Seq Profiling Analysis. *PLoS Comput Biol* **11**, e1004575 (2015). DOI 10.1371/journal.pcbi.1004575.
10. Zurauskiene, J. & Yau, C. pcareduce: hierarchical clustering of single cell transcriptional profiles. *BMC Bioinformatics* **17**, 140 (2016). DOI 10.1186/s12859-016-0984-y.
11. Kiselev, V. Y. *et al.* SC3: consensus clustering of single-cell RNA-seq data. *Nat Methods* **14**, 483–486 (2017). DOI 10.1038/nmeth.4236.
12. Lin, P., Troup, M. & Ho, J. W. K. CIDR: Ultrafast and accurate clustering through imputation for single-cell RNA-seq data. *Genome Biol* **18**, 59 (2017). DOI 10.1186/s13059-017-1188-0.
13. Trapnell, C. *et al.* The dynamics and regulators of cell fate decisions are revealed by pseudotemporal ordering of single cells. *Nat Biotechnol* **32**, 381–6 (2014). DOI 10.1038/nbt.2859.
14. Haghverdi, L., Buettner, F. & Theis, F. J. Diffusion maps for high-dimensional single-cell analysis of differentiation data. *Bioinformatics* **31**, 2989–98 (2015). DOI 10.1093/bioinformatics/btv325.
15. Setty, M. *et al.* Wishbone identifies bifurcating developmental trajectories from single-cell data. *Nat Biotechnol* **34**, 637–45 (2016). DOI 10.1038/nbt.3569.
16. van der Maaten, L. & Hinton, G. Visualizing data using t-SNE. *Journal of Machine Learning Research* **9**, 2579–2605 (2008).
17. Grün, D. *et al.* De Novo Prediction of Stem Cell Identity using Single-Cell Transcriptome Data. *Cell Stem Cell* **19**, 266–77 (2016). DOI 10.1016/j.stem.2016.05.010.
18. Vedaldi, A. & Soatto, S. Quick shift and kernel methods for mode seeking. In *European Conference on Computer Vision*, 705–718 (Springer, 2008).
19. van der Flier, L. G. & Clevers, H. Stem cells, self-renewal, and differentiation in the intestinal epithelium. *Annual review of physiology* **71**, 241–60 (2009). DOI 10.1146/annurev.physiol.010908.163145.
20. Barker, N. Adult intestinal stem cells: critical drivers of epithelial homeostasis and regeneration. *Nat Rev Mol Cell Biol* **15**, 19–33 (2014). DOI 10.1038/nrm3721.
21. Paul, F. *et al.* Transcriptional heterogeneity and lineage commitment in myeloid progenitors. *Cell* **163**, 1663–1677 (2015).
22. Weinreb, C., Wolock, S. & Klein, A. M. SPRING: a kinetic interface for visualizing high dimensional single-cell expression data. *Bioinformatics* **34**, 1246–1248 (2018). DOI 10.1093/bioinformatics/btx792.
23. Ji, Z. & Ji, H. TSCAN: Pseudo-time reconstruction and evaluation in single-cell RNA-seq analysis. *Nucleic Acids Res* **44**, e117 (2016). DOI 10.1093/nar/gkw430.
24. Rodriguez-Fraticelli, A. E. *et al.* Clonal analysis of lineage fate in native haematopoiesis. *Nature* **553**, 212–216 (2018). DOI 10.1038/nature25168.

25. Laurenti, E. & Gottgens, B. From haematopoietic stem cells to complex differentiation landscapes. *Nature* **553**, 418–426 (2018). DOI 10.1038/nature25022.

26. Nestorowa, S. *et al.* A single-cell resolution map of mouse hematopoietic stem and progenitor cell differentiation. *Blood* **128**, e20–31 (2016). DOI 10.1182/blood-2016-05-716480.

27. Herman, J. S., Sagar & Grun, D. FateID infers cell fate bias in multipotent progenitors from single-cell RNA-seq data. *Nat Methods* **15**, 379–386 (2018). DOI 10.1038/nmeth.4662.

28. Qiu, X. *et al.* Reversed graph embedding resolves complex single-cell trajectories. *Nat Methods* **14**, 979–982 (2017). DOI 10.1038/nmeth.4402.

29. Guyon, I., Weston, J., Barnhill, S. & Vapnik, V. Gene selection for cancer classification using support vector machines. *Machine learning* **46**, 389–422 (2002).

30. Bottou, L. Large-scale machine learning with stochastic gradient descent. In *Proceedings of COMPSTAT'2010*, 177–186 (Springer, 2010).

31. Pedregosa, F. *et al.* Scikit-learn: Machine learning in Python. *Journal of Machine Learning Research* **12**, 2825–2830 (2011).

32. Kamada, T. & Kawai, S. An algorithm for drawing general undirected graphs. *Information processing letters* **31**, 7–15 (1989).

33. Cox, D. R. The regression analysis of binary sequences. *Journal of the Royal Statistical Society. Series B (Methodological)* 215–242 (1958).

34. Fan, R.-E., Chang, K.-W., Hsieh, C.-J., Wang, X.-R. & Lin, C.-J. Liblinear: A library for large linear classification. *Journal of machine learning research* **9**, 1871–1874 (2008).

35. Pérez, F. & Granger, B. E. IPython: a system for interactive scientific computing. *Computing in Science and Engineering* **9**, 21–29 (2007). URL `http://ipython.org`. DOI 10.1109/MCSE.2007.53.
